# HPLC-ESI-MS/MS Profiling of Polyphenolics of a Leaf Extract from *Alpinia zerumbet* (Zingiberaceae) and Its Anti-Inflammatory, Anti-Nociceptive, and Antipyretic Activities In Vivo

**DOI:** 10.3390/molecules23123238

**Published:** 2018-12-07

**Authors:** Mosad A. Ghareeb, Mansour Sobeh, Samar Rezq, Assem M. El-Shazly, Mona F. Mahmoud, Michael Wink

**Affiliations:** 1 Medicinal Chemistry Department, Theodor Bilharz Research Institute, Kornaish El-Nile, Warrak El-Hadar, Imbaba (P.O. 30), Giza 12411, Egypt; 2 Institute of Pharmacy and Molecular Biotechnology, Heidelberg University, 44883-2462 Heidelberg, Germany; 3 Department of Pharmacology and Toxicology, Faculty of Pharmacy, Zagazig University, Zagazig 44519, Egypt; Samar_rezq@yahoo.com (S.R.); mona_pharmacology@yahoo.com (M.F.M.); 4 Department of Pharmacognosy, Faculty of Pharmacy, Zagazig University, Zagazig 44519, Egypt; assemels2002@yahoo.co.uk

**Keywords:** *Alpinia zerumbet*, antioxidant, anti-inflammatory, analgesia, antipyretic, HPLC-ESI-MS/MS

## Abstract

Reactive oxygen species (ROS) have been linked to several health conditions, among them inflammation. Natural antioxidants may attenuate this damage. Our study aimed to investigate the chemical composition of a methanol leaf extract from *Alpinia zerumbet* and its possible antioxidant, anti-inflammatory, anti-nociceptive, and antipyretic effects. Altogether, 37 compounds, representing benzoic and cinnamic acid derivatives and flavonoids (aglycones and glycosides), were characterized. The extract showed substantial in vitro antioxidant effects, and inhibited both cyclooxygenase 1 (COX-1) and cyclooxygenase 2 (COX-2) in vitro, with a higher selectivity towards COX-2. It also inhibited 5-lipoxygenase (LOX) activity in vitro with nearly double the potency of zileuton, a reference 5-lipoxygenase (LOX) inhibitor. The extract exhibited anti-inflammatory effects against carrageenan-induced rat hind paw edema, and suppressed leukocyte infiltration into the peritoneal cavity in carrageenan-treated mice. Furthermore, it possessed antipyretic effects against fever induced by subcutaneous injection of Brewer’s yeast in mice. Additionally, the extract demonstrated both central and peripheral anti-nociceptive effects in mice, as manifested by a decrease in the count of writhing, induced with acetic acid and an increase in the latency time in the hot plate test. These findings suggest that the leaf extract from *Alpinia zerumbet* could be a candidate for the development of a drug to treat inflammation and ROS related disorders.

## 1. Introduction

Overproduction of reactive oxygen species (ROS) is the major cause of oxidative stress. This phenomenon arises as a result of an imbalance between the level of ROS and the endogenous enzymatic antioxidants in the human body, and is followed by a series of health disorders, especially cancer, cardiovascular and liver diseases, and inflammation. ROS can oxidize the DNA base guanosine and thus induce point mutations [1,2].

Medicinal plants have a long history in the treatment of health disorders due to their diverse chemical composition of secondary metabolites. Among them, polyphenols are used as naturally occurring antioxidant agents due to their unique aromatic structure with a pronounced hydroxylation pattern [3].

*Alpinia zerumbet* (Pers.) B. L. Burtt and R. M. Smit (Zingiberaceae), commonly known as shell ginger, is native to East Asia and is presently being cultivated in tropical and subtropical zones worldwide. *A. zerumbet* has been used for a long time in folk and traditional medicine, for the treatment of many diseases and health disorders [4,5]. Several bioactive secondary metabolites were reported in *A. zerumbet*, including Kava lactones such as dihydro-5,6-dehydrokawain and 5,6-dehydrokawain as well as flavonoids, namely: rutin, kaempferol-3-*O*-rutinoside, and kaempferol-3-*O*-glucuronide and aliphatic homopolycyclic compounds, among others [6,7,8,9].

Furthermore, a broad spectrum of biological activities has been reported from different plant parts, including anti-nociceptive, antioxidant, hepatoprotective, cytotoxic, and antibacterial properties [8,10,11,12,13,14]. In addition, de Moura et al. [15] reported promising anti-hypertensive activities of leaves from plants grown in Brazil. As for the essential oil, dihydro-5,6-dehydrokawain and methyl cinnamate were the main component in rhizome oil, whereas 1,8-cineol, methyl cinnamate, and camphor dominated in the leaf oil from plants grown in Japan. On the other hand, monoterpenes (4-terpineol, 1,8-cineole and γ-terpinolene) dominated the essential oils from Japanese and Brazilian species [7,8,16].

The present work was undertaken to evaluate the in vitro antioxidant activities of leaves from *A. zerumbet* plants cultivated in Egypt, and to investigate its anti-inflammatory, anti-nociceptive, and antipyretic activities in animals. Furthermore, the chemical constituents of a methanol extract were characterized using HPLC-ESI-MS/MS.

## 2. Results

### 2.1. Identification of the Polyphenolics in a Methanol Extract from A. Zerumbet Leaves 

**Figure 1:**
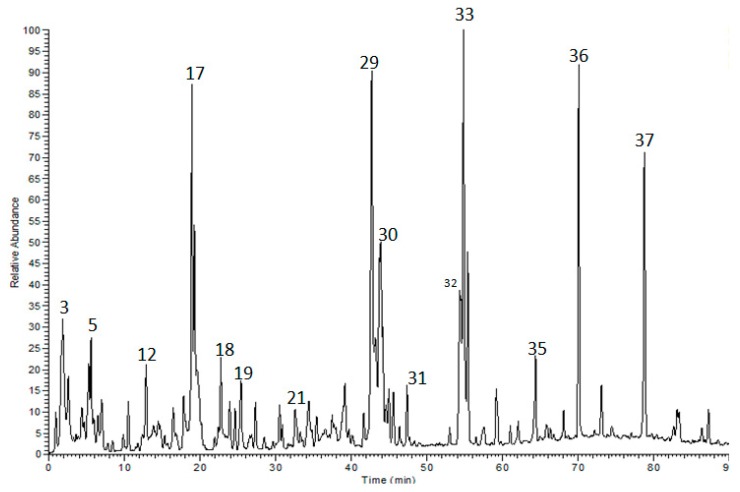
Negative HPLC-ESI-MS/MS profile of phenolic compounds from a methanol extract of *Alpinia zerumbet* leaves. Numbers at peaks refer to Table 1.

The polyphenolic compounds of the methanol extract from *A. zerumbet* leaves were identified via HPLC-ESI-MS/MS. 37 compounds were characterized, which consist of flavonoids (aglycones and glycosides) and benzoic and cinnamic acid derivatives. Table 1 summarizes the list of the compounds. Figure 1 shows the corresponding HPLC-ESI-MS/MS profile.

**Figure 2:**
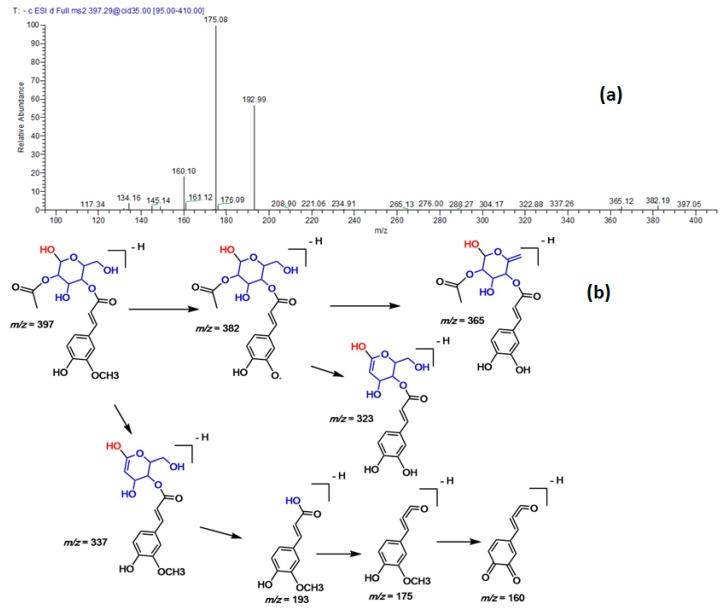
(**a**) MS/MS spectra of ferulic acid acyl-glucoside at [M − H]^−^ *m/z* 397. (**b**) A proposed fragmentation pattern in the negative ion mode.

**Figure 3:**
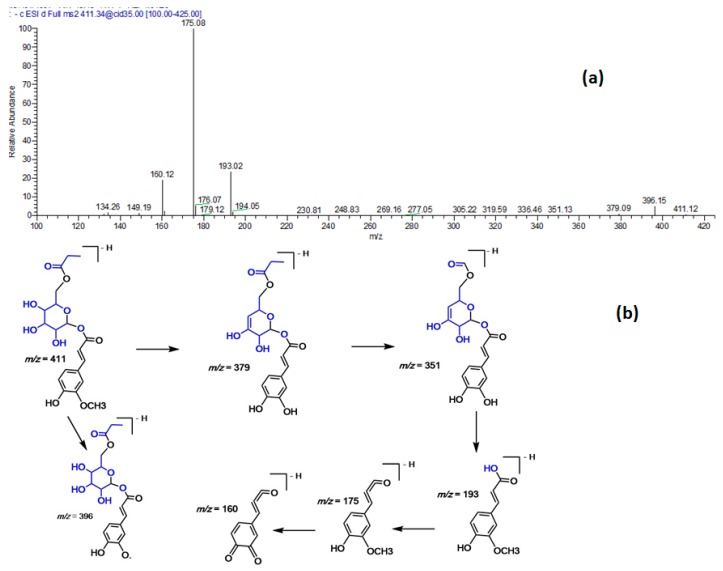
(**a**) MS/MS spectra of ferulic acid propionyl-glucoside at [M − H]^−^ *m/z* 411. (**b**) A postulated fragmentation pattern in the negative ion mode.

**Figure 4:**
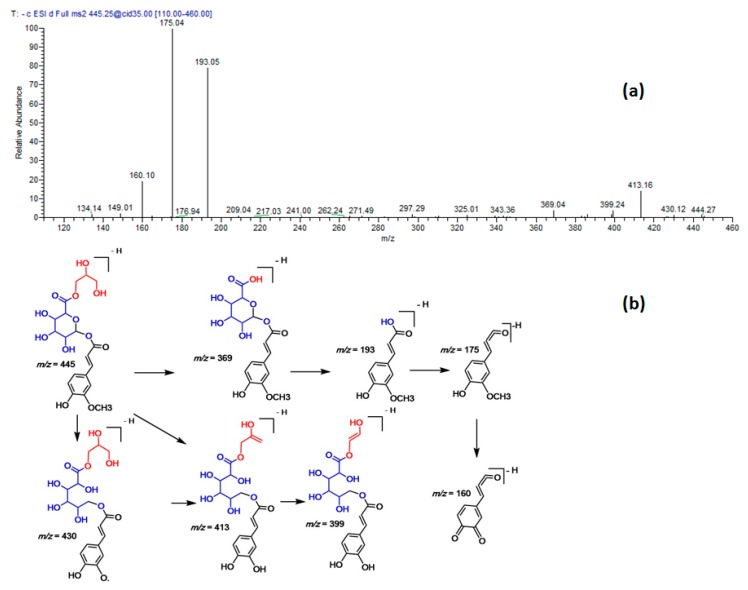
(**a**) MS/MS spectra of ferulyl *O*-glyceryl glucuronic acid at [M − H]^−^ *m/z* 445. (**b**) A postulated fragmentation pattern in the negative ion mode.

Several phenolic acids were reported in the extract. Among them, three new compounds were tentatively annotated based on their retention times, molecular weights, and MS/MS fragmentation. For instance, compound 22 exhibited a molecular ion peak at [M − H]^−^ *m/z* 397, and main fragment ions at 193, 175 and 160. The latter fragments are characteristic of ferulic acid, thus, the compound was assigned to ferulic acid acyl-glucoside (Figure 2). Similarly, compound 30 demonstrated a [M − H]^−^ *m/z* at 411, and main fragment ions at 193, 175, and 160. The difference between compound 22 and 30 was 14 amu (methyl group), thus, the latter was assigned to ferulic acid propionyl-glucoside (Figure 3). In analogy, two compounds (31 and 32) were identified as ferulyl *O*-glyceryl glucuronic acid isomers (Figure 4).

### 2.2. Antioxidant Activities

The extract exhibited pronounced antioxidant activities in vitro in three assays, namely DPPH, FRAP, and ABTS (Table 2). Furthermore, the extract exhibited TAC activity similar to that of ascorbic acid, used as an antioxidant standard (Table 2**)**.

### 2.3. Inhibition of Cyclooxygenase (COX1/2) and Lipoxygenase (LOX)

Initially, we investigated the ability of the extract to inhibit both COX-1 and COX-2 in vitro, to find out whether the extract would exhibit anti-inflammatory activities or not. Interestingly, the extract exhibited promising activities, and the inhibition of COX-1 by the extract was comparable to that of diclofenac. The extract inhibited COX-2 with half the potency of celecoxib. Moreover, the extract inhibited LOX in vitro with nearly double the potency of zileuton, the reference LOX inhibitor (Table 3).

### 2.4. Effects of the Extract on Carrageenan-Induced Paw Edema in Rats

**Figure 5:**
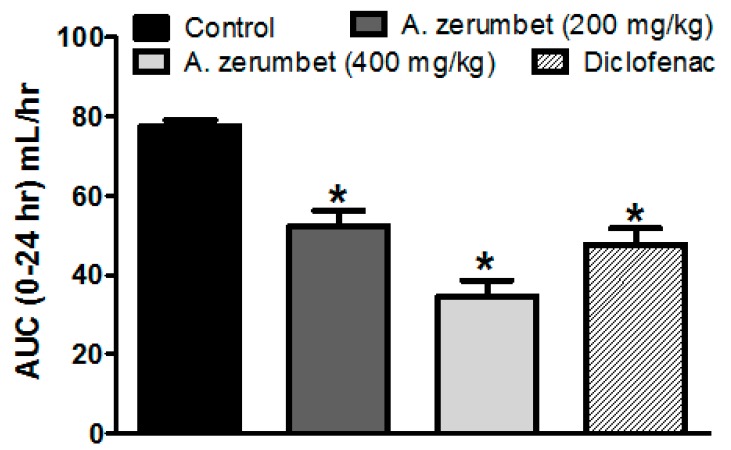
Effect of treatment with two dose levels (200 and 400 mg/kg, p.o.) of *A. zerumbet* or the reference standard, diclofenac (20 mg/kg, p.o.), on carrageenan-induced (1% suspension, 0.1 mL/rat) hind paw edema in rats. Edema thickness (mm) was measured before and every hour for 5 h, and at 24 h after carrageenan injection. Results represent the AUC_0-24_ after 24 h and are expressed as mean ± S.E.M. (n = 5). * *p* < 0.001 vs. control values.

Rats’ hind paws were challenged with 0.1 mL carrageenan (1% in 0.9%, sub-planter). They showed an inflammatory reaction, as recorded by an increase in paw thickness measured hourly for 5 h and then at 24 h post injection. The increase peaked at 4 h post injection to reach 4.1 ± 0.26 mm over baseline readings (paw thickness measured before carrageenan injection). On the other hand, rats pretreated 1 h earlier with the extract (200 and 400 mg/kg, p.o.) dose-dependently attenuated the increase in edema thickness values, represented as a decrease in the area under the time curve (AUC_0-24_), by 33 and 55% of control values, respectively (Figure 5). Additionally, the latter response was more pronounced than that achieved in rats treated with the standard anti-inflammatory drug, diclofenac (20 mg/kg, p.o.), which showed only a 40% reduced edema thickness compared to control rats.

### 2.5. Effects of the Extract on Carrageenan-Induced Leukocyte Migration into The Peritoneal Cavity in Mice

**Figure 6:**
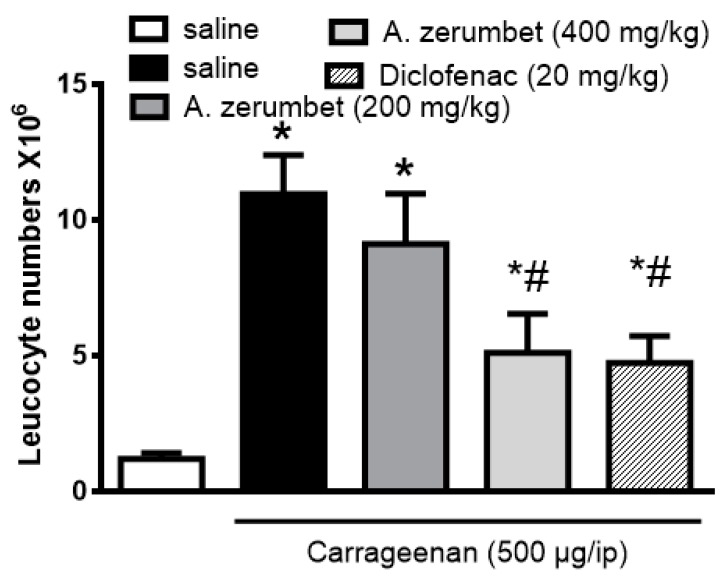
Effect of *A. zerumbet* extract in a dose of 200 and 400 mg/kg, p.o or diclofenac (20 mg/kg, p.o.) administration one hour before carrageenan injection (500 µg, i.p.) on leukocyte migration into the peritoneal cavity of mice (total number × 10^6^). Data is presented as mean ± S.E.M. (n = 5–6). * *p* < 0.01 vs. vehicle (saline) values, ^#^ *p* <0.01 vs. control (carrageenan treated group).

As shown in Figure 6, treatment of mice with the extract (200 and 400 mg/kg, p.o.) 1 h before carrageenan injection dose dependently attenuated carrageenan induced (500 μg/cavity, i.p., 0.1 mL) leukocyte migration into the peritoneal cavity by 34 and 48%, respectively. The effect of the highest dose (400 mg/kg, p.o.) was comparable to that of diclofenac (20 mg/kg, p.o.), which reduced total leukocyte count by 54% (Figure 6).

### 2.6. Effects of the Extract on Acetic Acid-Induced Vascular Permeability in Mice

**Figure 7:**
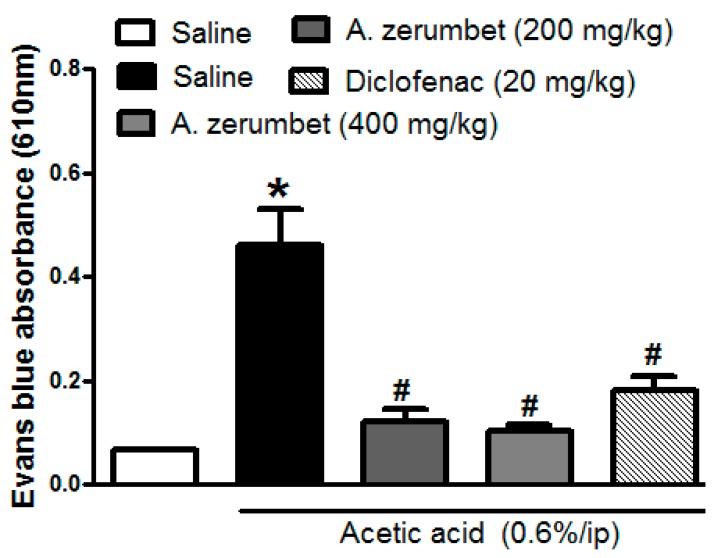
Effect of treatment with the extract (200 and 400 mg/kg, p.o.) on acetic acid-induced vascular permeability. The indicator of inflammation is the amount of Evans blue dye in the abdominal cavity. The values are expressed as the means ± S.E.M. (n = 5–7). * *p* < 0.001 compared to saline group. ^#^ *p* < 0.001 compared to control (acetic acid only treated group).

Acetic acid injection in mice (1 mL/100 g, 0.6%, i.p.) significantly increased (*p* < 0.001) Evans blue absorption in the peritoneal cavity exudate, which was utilized as a marker of vascular permeability. This increase was almost 7-fold as compared to saline injected controls. This effect was attenuated by pre-treating the mice with the extract (200 and 400 mg/kg, p.o.) 1 h prior to acetic acid injection by 74 and 78%, respectively. Additionally, the reference standard, diclofenac, achieved a 60% lower reading compared to control mice (Figure 7).

### 2.7. Effects of the Extract on Acetic Acid-Induced Writhing in Mice

**Figure 8:**
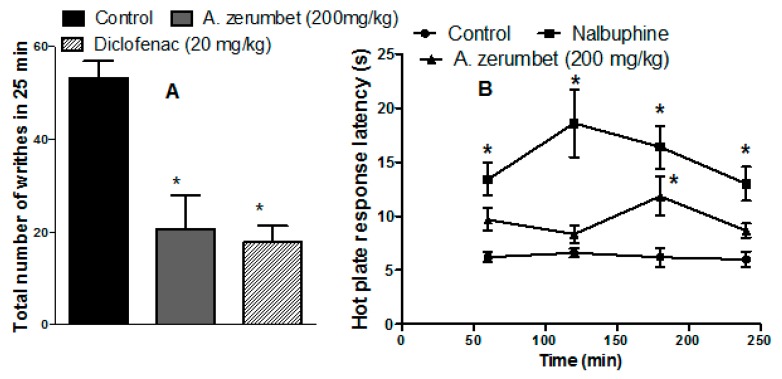
(**A**) Effect of treatment with *A. zerumbet* extract in a dose of 200 mg/kg, p.o., or diclofenac (20 mg/kg, p.o.) on acetic acid-induced writhing (0.7%, 1 mL/100 g) in mice. (**B**) Hot plate response latency (s) detected 1–4 h following administration of vehicle, extract (200 mg/kg, p.o.) or the reference standard, nalbuphine (10 mg/kg, p.o). Data is presented as mean ± S.E.M. (n = 5–8). * *p* < 0.05 vs. control values.

As shown in Figure 8A, injection of acetic acid (0.7% acetic acid, 1 mL/100 g) in mice resulted in a painful stimulus that appeared as abdominal writhes. Mice pre-treated with the extract (200 mg/kg, p.o.) showed a significantly lower number of writhes when observed over a period of 30 min (20.67 ± 2.33 vs. 53.25 ± 3.6) compared to controls. Additionally, mice pre-treated with diclofenac (20 mg/kg, p.o.) significantly (*p* < 0.001) inhibited the writhing response by 66% compared to vehicle treated mice (Figure 8A).

### 2.8. Anti-Nociceptive Effects of the Extract in Mice on Hot Plates

Animals pretreated with the extract and the reference anti-nociceptive drug, nalbuphine (10 mg/kg, i.p.), had longer response latency when measured at 1, 2, 3, and 4 h after administration. The effect was significant (*p* < 0.001) at all time points for nalbuphine, whereas the effect of the extract was only significant at 3 h post treatment, whereas it increased the latency period 1.9-fold over the control value (Figure 8B).

### 2.9. Antipyretic Effect of the Extract in Mice Injected with Brewer’s Yeast 

As shown in Table 4, mice injected with 30% Brewer’s yeast suspension (1 mL/100 g, s.c.) showed an increased rectal body temperature (38.36 ± 0.1) when measured 18 h post injection. Mice treated with the extract (200 mg/kg, p.o.) did not show any significant changes in rectal temperature over the whole period of experiment, which lasted 24 h. However, increasing the dose of the extract to 400 mg/kg showed fast and significant reduction in rectal temperatures compared to vehicle treated mice, starting 30 min post treatment (*p* < 0.01) with extended antipyretic action for up to 24 h. Notably, the latter effect was even stronger than that obtained with paracetamol (150 mg/kg), the reference antipyretic standard drug, which started to exert antipyretic effect 2 h posttreatment (Table 4).

## 3. Discussion

In this work, the phytochemical profile of a methanol extract from *A. zerumbet* leaves was comprehensively characterized using HPL-ESI-MS/MS. A total of 37 secondary metabolites were characterized. In addition to common phenolic acids such as cinnamic acid, isoferulic acid, eucomic acid, protocatechuic acid, vanillic acid, and sinapic acid, four new ferulic acid derivatives were tentatively annotated. Additionally, several flavonoids, among them apigenin, pinocembrin, kaempferol-3-*O*-*β*-d-glucuronide (previously reported from the plant), and genistein were characterized. However, rutin, catechin, and epicatechin were not detected in the current study [6], and this might be attributed to the geographical origin.

In addition, this study showed that the extract possessed potent antioxidant properties, free radical scavenging activity, and ferric reducing antioxidant power. Furthermore, the total antioxidant capacity (TAC) of the extract was similar to that of ascorbic acid, a well-known direct antioxidant. Previous studies showed that the essential oils from the leaves of *A. zerumbet* have potent antioxidant effects, in accordance with our study [17]. The antioxidant effects of the extract may be attributed to the phenolic acids and flavonoids content of the extract, which possess a potent antioxidant potential.

Free radical generation is implicated in the development of inflammation. Many inflammatory disorders can be alleviated by using antioxidants. The studied extract exhibited anti-inflammatory effects against carrageenan induced rat hind paw edema in the two dose levels (200 and 400 mg/kg). The effect of the highest dose level seems more potent, however, not significantly different from that of diclofenac, a well-known anti-inflammatory drug. 

We also tried to confirm the anti-inflammatory properties of the leaf extract, and to investigate its mechanism of action. Therefore, we used another model of inflammation, the carrageenan-induced leukocyte migration into peritoneal cavity in mice. The extract exerted a dose-dependent inhibitory effect against carrageenan induced leukocyte migration into the peritoneal cavity in mice. The highest used dose (400 mg/kg, p.o.) exerted a similar effect to that of diclofenac. 

Furthermore, the extract was more potent on acetic acid induced vascular permeability in mice than diclofenac. In this model, acetic acid causes an increase in the level of histamine in peritoneal fluids, serotonin, and prostaglandins, which leads to vasodilation of blood vessels and an increase in vascular permeability in the peritoneal cavity [18]. The current study indicates that the extract can prevent vasodilation and inhibit the release of inflammatory mediators in the acute vascular phase of inflammation. Different compounds from *A. zerumbet* fruit essential oil showed anti-inflammatory effects against dimethylbenzene-induced ear edema in rats when compared to aspirin [19].

Carrageenan-induced rat hind paw edema occurs in two phases, an early vascular phase and a late cellular phase, in which different mediators operate sequentially to produce the inflammatory response. Histamine, serotonin, and bradykinin represent the first detectable mediators in the early phase of carrageenan-induced inflammation, while the release of prostaglandins (PGs) is characteristic of the late phase of inflammation [20]. In the present study, the extract was capable of inhibiting COX-1 and thus the production of PGs. These results would explain the ability of the extract to subside carrageenan-induced rat hind paw edema. It was reported recently that the second accelerating phase of edema was not only attributed to the increased production of PGs, but also correlated to the induction of COX-2 in the hind paw. Therefore, we studied the effect of the extract on COX-2. The extract has a potent inhibitory effect on COX-2, and its effect was comparable to celecoxib, a well-known COX-2 inhibitor.

During the acute cellular phase of inflammation, infiltration of leukocytes, particularly neutrophils, from the blood into the tissue occurs. These neutrophils release oxygen-derived free radicals, among them hydroxyl radicals, superoxide anions (O^2−^), and other inflammatory mediators [20]. Another suggested mechanism by which the extract could inhibit the inflammatory response to carrageenan is the inhibition of the cellular phase by reducing the recruitment of neutrophil to the inflammation site. This idea was supported by the ability of the extract to decrease the number of leucocytes in the peritoneum of mice challenged with carrageenan. The previous effect may be attributed to the free radical scavenging power of the extract and the potent antioxidant activity of polyphenols [21].

Fever is a thermoregulatory indication of systemic inflammation [22]. Brewer’s yeast-induced pyrexia is caused by increasing the synthesis of prostaglandins, and therefore is used as a common model for the screening of active antipyretic drugs from natural and synthetic origins [23]. The present study showed that the high dose level of the extract significantly attenuated rectal temperature of yeast-induced febrile mice, and its effect was even more potent than acetaminophen (150 mg/kg), the well-known antipyretic drug. The effect of the extract may be attributed to the inhibition of prostaglandin synthesis in the hypothalamus. This was also confirmed by the ability of the extract to inhibit both COX-1 and COX-2 in our in vitro study.

The present study also investigated the anti-nociceptive activity of the extract by two methods. These methods included chemical nociception in the test model of acetic acid-induced writhing, and thermal nociception in the hot plate test. The hot plate test is a typical model to evaluate narcotic analgesia or central anti-nociceptive effects. The anti-nociceptive potential of the extract was shown by hot plate test (significant only 3 h post treatment), while nalbuphine, the narcotic anti-nociceptive standard, had a significant effect at all time points. It is assumed that the extract has a peripheral anti-nociceptive effect, but a less potent central anti-nociceptive effect than nalbuphine.

The writhing test is often used to evaluate the peripheral anti-nociceptive activity of drugs. Acetic acid causes secretion of endogenous pain mediators, thus stimulating the neurons responsible for pain sensation [24]. In this study, the low dose level of the extract demonstrated solid anti-nociceptive effects in the acetic acid-induced writhing test in mice. This result suggests that the extract has peripheral anti-nociceptive properties and they might be attributed by blocking the release of endogenous inflammatory mediators such as leukotriene, prostaglandin, serotonin, and histamine. This assumption was supported by the ability of the extract to inhibit both COX and LOX in the in vitro study. The biological activity data suggest that the anti-inflammatory and different anti-nociceptive effects of the *A. zerumbet* extract are directly linked to its flavonoids content such as quercetin 3-*O*-glucoside, isorhamnetin 3-*O*-rutinoside, kaempferol-3-*O*-β-d-glucuronide, apigenin, chrysoeriol, and diosmetin as well as its phenolic acids composition, among them gallic acid, cinnamic acid, eucomic acid, vanillic acid 4-β-d-glucoside, and protocatechuic acid [21].

A previous study by de Araújo et al. [11] was in accordance with our findings. They showed that orally administered essential oil of *A. zerumbet*, at doses of 100 mg/kg and 300 mg/kg, had anti-nociceptive effects against acetic acid-induced writing, formalin-induced paw licking effect, and increased the latency in the hot plate test. They also showed that the central anti-nociceptive effect in the hot plate test was partially mediated through opiate receptors, as it was partially blocked by naloxone, an opioid antagonist.

In summary, we conclude that the polyphenol-rich *A. zerumbet* extract can exert anti-nociceptive, antipyretic, and anti-inflammatory effects by scavenging of free radicals from the site of injury and inhibiting the synthesis of prostaglandins and leukotrienes.

## 4. Materials and Methods

### 4.1. Plant Material and Extraction

Fresh leaves were collected from Zoo Garden, Giza, Egypt in May 2014. Plant identity was confirmed by Dr. Tearse Labib, Department of Flora and Taxonomy, El-Orman Botanical Garden, Giza, Egypt. Voucher specimen (No. A4/1/6) have been deposited in the herbarium of the garden. Dry powdered leaves (1.8 kg) were macerated in methanol at room temperature. The extract was concentrated using a rotatory evaporator (Buchi, Switzerland) at 40 ± 2 °C. The crude methanol extract (150 g) was defatted by petroleum ether (60–80 °C), yielding 115 g after freeze-drying. 

### 4.2. HPLC-ESI-MS/MS Conditions

HPLC-ESI-MS/MS was employed to investigate the chemical constituents of the extract. The LC system was Thermo Finnigan (Thermo electron Corporation, USA), coupled with an LCQ Duo ion trap mass spectrometer with an ESI source (ThermoQuest). A Silica gel C18 reversed-phase column (Zorbax Eclipse XDB-C18, Rapid resolution, 4.6 × 150 mm, 3.5 µm, Agilent, USA) was used for the separation process. Water with a gradient increase from 5% to 50% of acetonitrile (ACN) (with 1% formic acid each in the positive mode) was applied in 60 min, with a flow rate 1 mL/min, and then increased to 90% ACN in the next 30 min. The samples were injected automatically using auto sampler surveyor ThermoQuest. The instrument was controlled by Xcalibur software. The MS operating conditions were applied in the negative ion mode, as previously described by us [25]. The ions were detected in a full scan mode and mass range of 50–2000 *m/z*.

### 4.3. Total Phenolic Content (TPC) and Antioxidant Assays

TPC of the investigated extract was determined by the Folin-Ciocalteu assay [26]. DPPH radical scavenging assay, ferric reducing antioxidant power assay (FRAP), and ABTS radical-scavenging activity assay were carried out in triplicate as described in Ghareeb et al. [26]. The total antioxidant capacity (TAC) was investigated using a commercially available ELISA kit (MBS726896, mybiosource, Inc., San Diego, CA, USA) according to the manufacturer’s instructions [27].

### 4.4. In Vitro Anti-Inflammatory Studies

#### Cyclooxygenase (COX) and Lipoxygenase (LOX) Inhibition Assays

Inhibition of bovine COX-1 and COX-2 by *A. zerumbet* extract was determined in vitro using an enzyme immuno assay (EIA) kit (Cayman Chemical, AnnArbor, MI, USA) [28]. A lipoxygenase inhibitor screening assay kit (Cayman Chemical, AnnArbor, MI, USA) was used to evaluate lipoxygenase inhibition activity [28].

### 4.5. In Vivo Anti-Inflammatory Studies

All animals were obtained from the Faculty of Veterinary Medicine, (Zagazig, Egypt), and acclimatized to the experimental conditions for one week before starting each experiment. Animals were housed in a light-controlled room with a 12 h light/dark cycle and constant ambient humidity. They were allowed free access to food and water. All experimental procedures and animal care methods in this study were approved by Ethical Committee of the Faculty of Pharmacy, Zagazig University for Animal Use (Zagazig, Egypt).

#### 4.5.1. Anti-Inflammatory Activity in Carrageenan-Induced Hind-Paw Edema in Rats

The carrageenan-induced paw edema model is a well-established model for investigating novel anti-inflammatory candidates [29,30]. The vehicle (10 mL/kg), *A. zerumbet* extract (200 and 400 mg/kg, p.o.), or diclofenac (20 mg/kg, p.o) were given to the rats 1 h before carrageenan challenge. Next, carrageenan solution (1% in 0.9% NaCl, 0.1 mL) was injected into the sub plantar tissue of the rat hind paw. The paw thickness (mm) was measured before and after the carrageenan injection, at hourly intervals for 5 h and then at 24 h, using a caliper ruler. The area under the changes in paw thickness–time curve for the whole observation period of 24 h was calculated to demonstrate the cumulative anti-inflammatory effect of the extract. 

#### 4.5.2. Recruitment of Leukocytes to Peritoneal Cavity in Mice

The ability of the extract to inhibit leukocyte recruitment to the peritoneal cavity after carrageenan injection was investigated as described previously [31]. Briefly, Swiss albino mice (average weight 25–30 g, n = 5–6/group), were treated with the vehicle (1 mL/100 g, p.o.) as a control group, or the extract in two different doses (200 and 400 mg/kg, p.o.), or the standard compound diclofenac (20 mg/kg, p.o.). 30 min later, 0.1 mL carrageenan solution (500 μg/mice) or 0.1 mL sterile saline was injected intraperitoneally. The animals were euthanized after 3  h, and the peritoneal cavity was rinsed using 3 mL of phosphate-buffered saline (PBS) containing 1 mM (EDTA). The total leukocyte count was determined in the peritoneal cavity wash using a hemocytometer, and expressed as number of cells/mL.

#### 4.5.3. Acetic Acid-Induced Vascular Permeability in Mice

The acetic acid-induced vascular permeability test was performed as previously described [32]. Briefly, mice were treated with the extract (200 and 400 mg/kg, p.o.), diclofenac (20 mg/kg), or vehicle. 1 h later, the tail vein was injected with 0.2 mL Evans blue (0.25% solution in normal saline). After 30 min, acetic acid (0.6% in normal saline, 1 mL/100 g) was injected in the peritoneal cavity of the mice. Another group of mice was injected with normal saline only, and served as a control. After another 30 min, the mice were sacrificed by cervical dislocation. The abdominal cavities were washed with 3 mL saline, and the washings were then centrifuged at 3000 rpm for 10 min. The vascular permeability is proportional to Evans blue dye content of the supernatant that was detected at 610 nm using a plate reader (BioTeK, Vt, USA).

#### 4.5.4. Anti-Nociceptive Activity by Hot Plate Test in Mice

Swiss albino mice weighing 25–30 g (n = 5–6/group) received the extract (200 mg/kg, p.o.), the vehicle (10 mL/kg, p.o.), or the reference compound nalbuphine (10 mg/kg, i.p.). After 1 h, the mice response to heat-induced nociceptive pain was assessed individually by placing on a hot plate heated at 55 ± 1 °C. The latency until mice showed first signs of discomfort (licking of the fore and hind paws, hind paw lifting, or jumping) was recorded, before (baseline) and at 1, 2, 3 and 4 h following different treatments.

#### 4.5.5. Induction of Pyrexia in Mice Using Brewer’s Yeast

Pyrexia was induced in mice as described previously, with modifications [22]. Briefly, initial body temperature of the rectum was recorded for each mouse using a lubricated digital thermometer. Next, Brewer’s yeast suspension was prepared in normal saline (30%), and then the yeast suspension (1 mL/100 g) was injected subcutaneously behind the neck. 18 h later, the rectal temperature was recorded again (T_0_), and mice that showed a temperature higher than T_0_ by at least 0.5 °C were included in the study. Pyretic animals were then treated with the extract, paracetamol (150 mg/kg) or vehicle. The rectal temperature was recorded again at 30 min, 1, 2, 3 and 24 h posttreatment.

#### 4.5.6. Anti-Nociceptive Activity by Acetic Acid-Induced Abdominal Writhing in Mice

The acetic acid-induced writhing model is commonly used to assess the peripheral anti-nociceptive activity of the drug under investigation [33]. Swiss albino mice weighing 25–30 g were divided into 3 groups (6–8 mice) and pretreated with the vehicle (1% Tween 80,10 mL/kg), the extract (200 mg/kg, p.o.), or diclofenac (20 mg/kg) for 1 h, followed by 0.7% acetic acid injection (1 mL/100 g, i.p.). The number of writhes (constriction of abdomen, turning of trunk and extension of hind legs) was recorded for 25 min.

### 4.6. Data Analysis

Data were analyzed using statistical software Graph Pad Prism version 5 (GraphPad Software, San Diego, CA, USA.). Analysis of Variance (ANOVA) or repeated-measures analysis of variance (RM-ANOVA), after which by Tukey’s post hoc test and Student’s *t*-test were used to state differences between groups. Data are expressed as mean ±  S.E.M.

## Figures and Tables

**Table 1:** Phenolic compounds in the methanol extract via negative HPLC-ESI-MS/MS.

No.	Rt	[M − H]^−^	MS/MS	Identified Compounds
1	1.11	191	173, 127, 111	Quinic acid ^a^
2	1.32	133	115	Malic acid ^a^
3	1.52	169	169, 125	Gallic acid ^a^
4	1.57	147	129, 115	Cinnamic acid ^a,b^
5	4.89	239	221, 193, 179	Eucomic acid ^a^
6	5.05	189	175, 157,129	3-Dehydroquinic acid ^a^
7	5.30	209	191	5-Hydroxyferulic acid ^a^
8	5.78	153	109, 97, 81	Protocatechuic acid ^a^
9	7.59	329	314, 209, 167, 123	Vanillic acid 4-β-d-glucoside ^a^
10	8.38	179	135, 133, 125, 107	Caffeic acid ^a^
11	9.92	137	93	Salicylic acid ^a^
12	12.81	167	151, 123, 109	Vanillic acid ^a,^^b^
13	14.02	197	182, 167, 153	Syringic acid ^a,b^
14	14.70	163	119	*p*-Coumaric acid ^a^^,b^
15	16.47	385	223	Sinapic acid 3-*O*-glucoside ^a^
16	17.66	441	397, 331, 330, 289	Epicatechin 3-*O*-gallate ^a^
17	18.94	223	205, 191, 163	Sinapic acid ^a^
18	22.97	355	193, 175, 160, 135	Isoferulic acid 3-*O*-β-glucopyranoside ^a^
19	24.26	193	193, 179, 149	Isoferulic acid ^a^
20	36.66	431	311, 281, 179, 151	Isovitexin ^a^
21	30.16	463	301, 179	Quercetin 3-*O*-glucoside ^a^
22	32.70	397	193, 175	Ferulic acid acyl-glucoside
23	34.28	623	315, 300, 255	Isorhamnetin 3-*O*-rutinoside ^a^
24	34.33	593	327, 285	Kaempferol 3-*O*-rutinoside ^a,c^
25	34.67	477	315, 299	Isorhamnetin 3-*O*-glucoside ^a^
26	35.32	447	285, 255, 151	Kaempferol 3-*O*-glucoside ^a^
27	36.45	433	301	Quercetin 3-*O*-pentoside ^a^
28	39.05	491	315, 300, 271, 179	Isorhamnetin-3-*O*-β-d-glucuronide ^a^
29	42.59	475	285, 271, 179	Kaempferol-3-*O*-β-d-glucuronide ^a,c^
30	44.55	411	193, 175	Ferulic acid propionyl-glucoside
31	47.25	445	413, 193, 175	Ferulyl *O*-glyceryl glucuronic acid
32	53.81	445	413, 193, 175	Ferulyl *O*-glyceryl glucuronic acid
33	54.14	269	269, 255	Apigenin ^a^
34	59.13	299	285	Chrysoeriol ^a^
35	64.16	299	284	Diosmetin ^a^
36	70.11	255	255, 213, 151	Pinocembrin ^a^
37	78.74	269	269	Genistein ^a^

^a^ Compounds were identified based on authentic compounds. ^b^ Previously identified in the plant using GC-MS and reference standards [8]. ^c^ Previously isolated from the plant [6].

**Table 2:** Total phenolic content (TPC) and in vitro antioxidant activities of the methanol extract of *A. zerumbet* leaves. Ascorbic acid, quercetin and trolox were used as positive controls.

Treatment	TPC	DPPH	ABTS	FRAP	TAC
	(mg GAE/g extract)	(IC_50_ µg/mL)		(mM FeSO_4_ equivalent/mg extract)	U/L
Extract	226.98 ± 9.84	23.54 ± 1.78	5.52 ± 0.34	13.97 ± 0.43	30.20 ± 1.23
Ascorbic acid	–	2.92 ± 0.29	–	–	27.12 ± 1.11
Quercetin	–	–	–	21.45 ± 2.55	–
Trolox	–	–	1.63 ± 0.46	–	–

DPPH: 2 2-diphenyl-1-picrylhydrazylfree radical scavenging assay. FRAP: Ferric reducing antioxidant power. ABTS: 2,2′-azino-bis(3-ethylbenzothiazoline-6-sulphonic acid radical-scavenging assay. TAC: Total antioxidant capacity. Data shown are the means ± S.D, n = 3).

**Table 3:** Effect of *A. zerumbet* extract on the activities of COX-1, COX-2, and LOX enzymes, calculated as extract concentration that caused 50% inhibition of enzyme activity (IC_50_). Celecoxib, diclofenac, indomethacin, and zileuton were used as positive controls.

Treatment	COX-1	COX-2	SI	LOX
	IC_50_ (µg/mL)			IC_50_ (µg/mL)
Extract	3.65 *^,@^ ± 0.45	0.078 ^@#^ ± 0.002	46.80	1.92 ** ± 0.37
Celecoxib	16.50 ± 1.17	0.046 ± 0.027	358.70	–
Diclofenac	4.20 ± 0.46	0.76 ± 0.18	5.52	2.31 ± 0.53
Indomethacin	0.042 ± 0.012	0.53 ± 0.20	0.08	–
Zileuton	–	–	–	3.21 ± 0.40

Data are presented as mean ± SD, n = 3. * significantly different from celecoxib, ^@^ significantly different from indomethacin, ^#^ significantly different from diclofenac, and **significantly different from zileuton at *p* < 0.05 by One-way analysis of variance (ANOVA) followed by Tukey post hoc test using GraphPad prism, version 5. SI is COX selectivity index, which is defined as IC_50_ (COX-1)/IC_50_ (COX-2).

**Table 4:** Effect of treatment with *A. zerumbet* extract in a dose of 200 and 400 mg/kg or paracetamol in a dose of 150 mg/kg on Brewer’s yeast induced pyrexia in mice.

Groups	Rectal Temperature ^#^	Rectal Temperature Recorded Following Different Treatments (°C)				
		30 min	1 h	2 h	3 h	24 h
Control	38.3 ± 0.2	38.4 ± 0.1	38.5 ± 0.1	38.9 ± 0.2	39 ± 0.3	38.3 ± 0.2
Extract (200 mg /kg)	38.5 ± 0.2	38.4 ± 0.1	38.4 ± 0.2	38.1 ± 0.2	38.3 ± 0.4	37.81 ± 0.3
Extract (400 mg /kg)	38.1 ± 0.3	36.6 ± 0.6 *	36.8 ± 0.7 *	36.8 ± 0.6 *	36.1 ± 0.3 *	36.7 ± 0.2 *
Paracetamol (150 mg/kg)	38.5 ± 0.2	38.1 ± 0.2	37.5 ± 0.3	37 ± 0.3 *	36.9 ± 0.2 *	36.3 ± 0.2 *

^#^ Rectal temperature was recorded at 18 h following yeast injection. Values are presented as mean ± S.E.M. (n = 5), * *p* < 0.001 vs. control values.
